# How to interact with medical terminologies? Formative usability evaluations comparing three approaches for supporting the use of MedDRA by pharmacovigilance specialists

**DOI:** 10.1186/s12911-020-01280-1

**Published:** 2020-10-09

**Authors:** Romaric Marcilly, Laura Douze, Sébastien Ferré, Bissan Audeh, Carlos Bobed, Agnès Lillo-Le Louët, Jean-Baptiste Lamy, Cédric Bousquet

**Affiliations:** 1 Univ. Lille, CHU Lille, ULR 2694 - METRICS: Évaluation des technologies de santé et des pratiques médicales, F-59000 Lille, France; 2 Inserm, CHU Lille, CIC-IT/Evalab 1403 – Centre d’Investigation Clinique, F-59000 Lille, France; 3 SemLIS - Semantics, Logics, Information Systems for Data-User Interaction, Rennes, France; 4 Laboratoire d’informatique médicale et d’ingénierie des Connaissances en e-santé, LIMICS, Sorbonne Université, Inserm, Université Paris 13, 75006 Paris, France; 5 Everis / NTT Data, University of Zaragoza, Zaragoza, Spain; 6 Centre Régional de Pharmacovigilance HEGP, AP-HP, Paris, France; 7 Université Sorbonne Paris Nord, LIMICS, INSERM, UMR 1142, F-93000 Bobigny, France; 8 SSPIM, Unit of Public Health and Medical Informatics, CHU University Hospital of Saint Etienne, Saint-Étienne, France

**Keywords:** Formative evaluation, Usability testing, Cognitive walkthrough, Pharmacovigilance, MedDRA

## Abstract

**Background:**

Medical terminologies are commonly used in medicine. For instance, to answer a pharmacovigilance question, pharmacovigilance specialists (PVS) search in a pharmacovigilance database for reports in relation to a given drug. To do that, they first need to identify all MedDRA terms that might have been used to code an adverse reaction in the database, but terms may be numerous and difficult to select as they may belong to different parts of the hierarchy. In previous studies, three tools have been developed to help PVS identify and group all relevant MedDRA terms using three different approaches: forms, structured query-builder, and icons. Yet, a poor usability of the tools may increase PVS’ workload and reduce their performance. This study aims to evaluate, compare and improve the three tools during two rounds of formative usability evaluation.

**Methods:**

First, a cognitive walkthrough was performed. Based on the design recommendations obtained from this evaluation, designers made modifications to their tools to improve usability. Once this re-engineering phase completed, six PVS took part in a usability test: difficulties, errors and verbalizations during their interaction with the three tools were collected. Their satisfaction was measured through the System Usability Scale. The design recommendations issued from the tests were used to adapt the tools.

**Results:**

All tools had usability problems related to the lack of guidance in the graphical user interface (e.g., unintuitive labels). In two tools, the use of the SNOMED CT to find MedDRA terms hampered their use because French PVS were not used to it. For the most obvious and common terms, the icons-based interface would appear to be more useful. For the less frequently used MedDRA terms or those distributed in different parts of the hierarchy, the structured query-builder would be preferable thanks to its great power and flexibility. The form-based tool seems to be a compromise.

**Conclusion:**

These evaluations made it possible to identify the strengths of each tool but also their weaknesses to address them before further evaluation. Next step is to assess the acceptability of tools and the expressiveness of their results to help identify and group MedDRA terms.

## Background

Medical terminologies are commonly used today in medicine. They are intended to implement semantic interoperability between the various information systems, and to support search in databases [1]. However, clinicians often have difficulties to use these terminologies during their practice [2]. In particular, terminologies may suffer from synonymy, polysemy, misclassification, poor coverage or, on the contrary, duplicated terms. The evolution of terminologies also makes them more and more complex, e.g. with the recent advance in genetics, as terminologies have to remain backward compatible. Few usability studies have specifically been focused on the use of medical terminologies when querying clinical data. A study showed a poor usability of a compositional interface terminology based on a subset of SNOMED CT [3]. Moreover, usability problems found in EHR frequently include terminology-related issues [4]. A particular case is the use of MedDRA (Medical Dictionary for Drug Regulatory Activities) by pharmacovigilance specialists (PVS).

Pharmacovigilance is “the science and activities relating to the detection, assessment, understanding and prevention of adverse effects or any other drug-related problem” [5]. PVS supervise and analyze the adverse drug reactions (ADR) reported by healthcare professionals (e.g., general practitioners, pharmacists) and patients to healthcare authorities or to the marketing authorization holder. Based on these spontaneous reports, they collect information on the reported ADR, the concerned drug, and the patients’ health conditions, contacting afterwards the reporter if more information is needed. Once all the data is gathered, a report synthetizing the event is registered into a pharmacovigilance database using coded terms that closely capture the original verbatim describing the event. For international standardization purposes, the International Council for Harmonization of Technical Requirements of Pharmaceuticals for Human Use has developed since 1996 a standardized medical terminology thesaurus, MedDRA, that is nowadays widely used and even mandatory in some countries for safety reports (e.g., in European Union countries) [6]. The MedDRA terminology enables to classify the ADR using a 5-level hierarchy of terms that are superordinate or subordinate to each other: from the broadest groups of terms to the more specific, the levels are System Organ Class (SOC), High-Level Group Terms (HLGT), High-Level Terms (HLT), Preferred Terms (PT), and Low-Level Terms (LLT) [7]. Depending on the level of specificity required, ADR are coded with more or less specificity using LLT terms.

Besides, PVS are also responsible for answering pharmacovigilance questions. Those questions may be asked by physicians, pharmacists or even patients who are worried about a symptom and wondering whether this symptom is an adverse reaction to an administered drug. Patients might also ask for advice on a treatment or on management of the ADR. To answer those questions, PVS search information in heterogeneous data sources: reference books (e.g. Martindale or DRUGDEX®), scientific literature (e.g. querying PubMed), summaries of product characteristics, and national and international pharmacovigilance databases (e.g., VigiBase, the WHO global database, or the French pharmacovigilance database, *banque nationale de pharmacovilance* - BNPV). Once the PVS gathers enough information to answer the question, the spontaneous report associated with the question is entered and coded in the pharmacovigilance database. Healthcare authorities may also have demands regarding signal detection and monitoring (i.e., enhanced monitoring of a drug to detect a previously unknown relation between an ADR and such a drug). In this case, PVS will perform a systematic and exhaustive search in the pharmacovigilance database for all case reports that were reported in relation to the concerned drug.

When PVS need to identify all reports related to a given type of ADR, they first need to identify all MedDRA terms that might have been used to code it in the pharmacovigilance database. Yet, several problems related to the coding of the ADR cases and to the structure of the MedDRA hierarchy hinder identifying and grouping MedDRA terms.

*Coding problems*. At the step of entering the case report in the database, the PVS’ profile (i.e. pharmacist or physician), experience, expertise in the type of ADR, expertise in MedDRA, and the initial description of the ADR (i.e., patient’s symptoms, details in the description, wording), may influence the choice of the MedDRA terms used to code the ADR. As a result, different MedDRA terms might be used to code the same ADR. For instance, “purpura” is a small hemorrhage of the skin, but also mucous membrane which may be caused by various factors. The term “purpura” or “ecchymoses” can be coded with very various terms some of which may pose problems of choice or diagnosis (ex. “purpuric rash”, “rash hemorrhagic”, “purpura symptomatica”, “Henoch-Schönlein”, “allergic vascular purpura”).

**Fig. 1:**
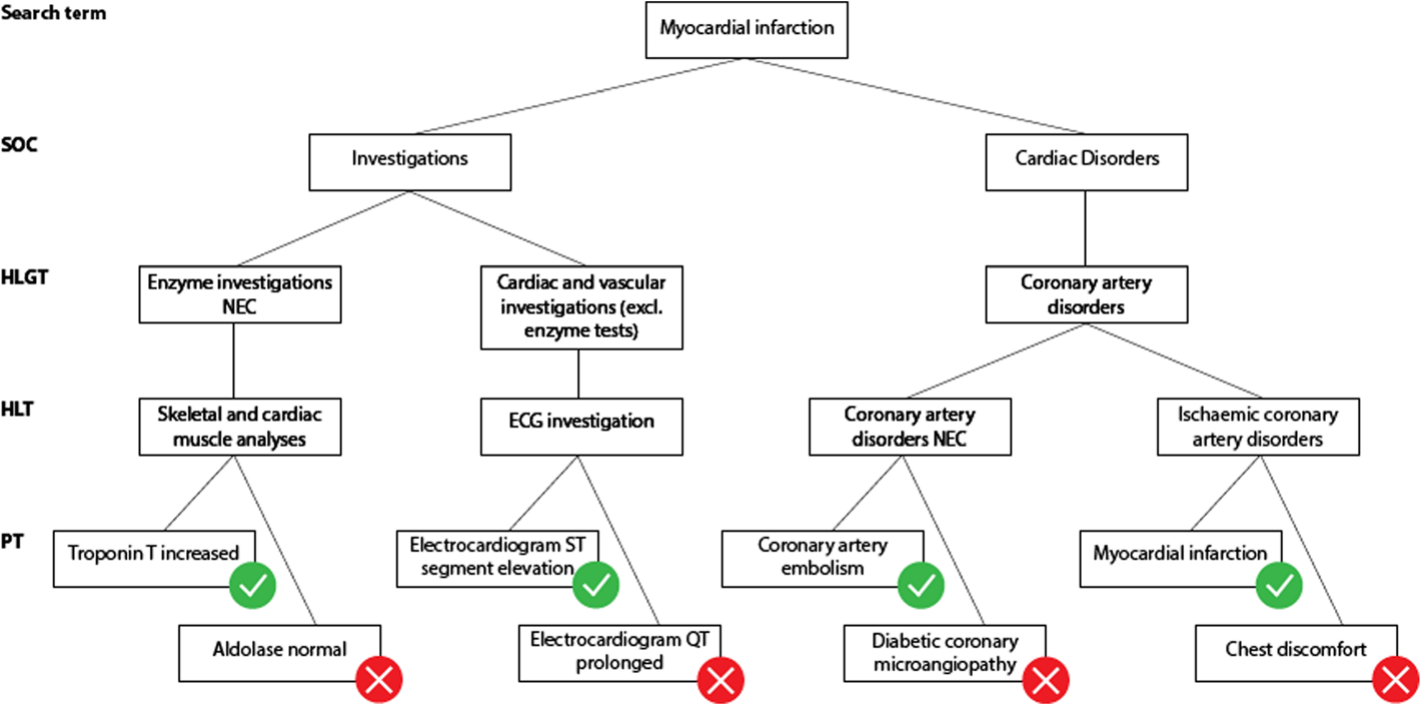
Illustration of the heterogeneity of the MedDRA categories used to code the ADR “Myocardial infarction”. Relevant PT (green circle) are found in two SOC (“investigations” and “cardiac disorders”). Some HLT categories contain both relevant and irrelevant (red circle) terms

*MedDRA structure problems*. Brown has described in 2003 several pitfalls that make it difficult to search case reports in pharmacovigilance databases with MedDRA [8]. Further evolutions of MedDRA have attempted, and in some cases succeeded, to lower such difficulties. Yet, terms representing a same medical condition may be scattered all over the MedDRA hierarchy, belonging to different SOC at different levels [9]. The MedDRA structure still makes it difficult to build custom groupings of MedDRA terms that are representative of a medical condition. For example (Fig. 1), when searching case reports of “Myocardial infarction” it is relevant to include terms that describe clinical conditions such as “Coronary artery embolism” but also results of investigations such as “Troponin T increased”. However, investigations are not linked to the corresponding medical conditions in MedDRA which does not enable to perform such association in an automated way. Besides, the structure of MedDRA does not systematically allow selecting a single category where all terms relevant to a given medical condition would be present excluding others. This is the case for “Myocardial infarction”. The HLGT “Coronary artery disorders” contains some relevant terms such as “Coronary artery embolism” or “Myocardial infarction” but also non relevant terms such as “Diabetic coronary microangiopathy” or “Chest discomfort”. The HLT “Skeletal and cardiac muscle analyses” includes the PT “Troponin T increased” that is evocative of myocardial infarction while the PT “Aldolase normal” is not. Besides, there is no intersection between “Troponin T increased” and “Electrocardiogram ST segment elevation” below SOC Investigations. It would therefore be useful for MedDRA to propose a greater number of groupings that would contain only the terms relevant to that grouping. However, in a given SOC, a PT can only belong to one HLGT. Thus, it is not possible to constitute in the same SOC several HLGT groups containing the same PT [8].

The current solution to these problems is to build Standardized MedDRA Queries (SMQ) that group PTs from different SOC, HLGT and HLT [10]. A SMQ is available for MedDRA users, but the number of SMQ is still insufficient to cover every need when querying a pharmacovigilance database, and new means to support building such groupings in a more automated way are desirable.

Tools linking ADR and MedDRA terms could help PVS in this task. This article presents the formative evaluations of three tools developed for this purpose in the PEGASE (*Pharmacovigilance Enrichie par des Groupements Améliorant la détection des Signaux Émergents* - in English, improved pharmacovigilance and signal detection with groupings) project. This project is a project funded by the French national research agency, and its goals are to develop usable tools to help PVS finding all relevant MedDRA terms that might have been used to code a given ADR, and to ensure searches achieve a good completeness.

## Study context: the PEGASE project

**Fig. 2:**
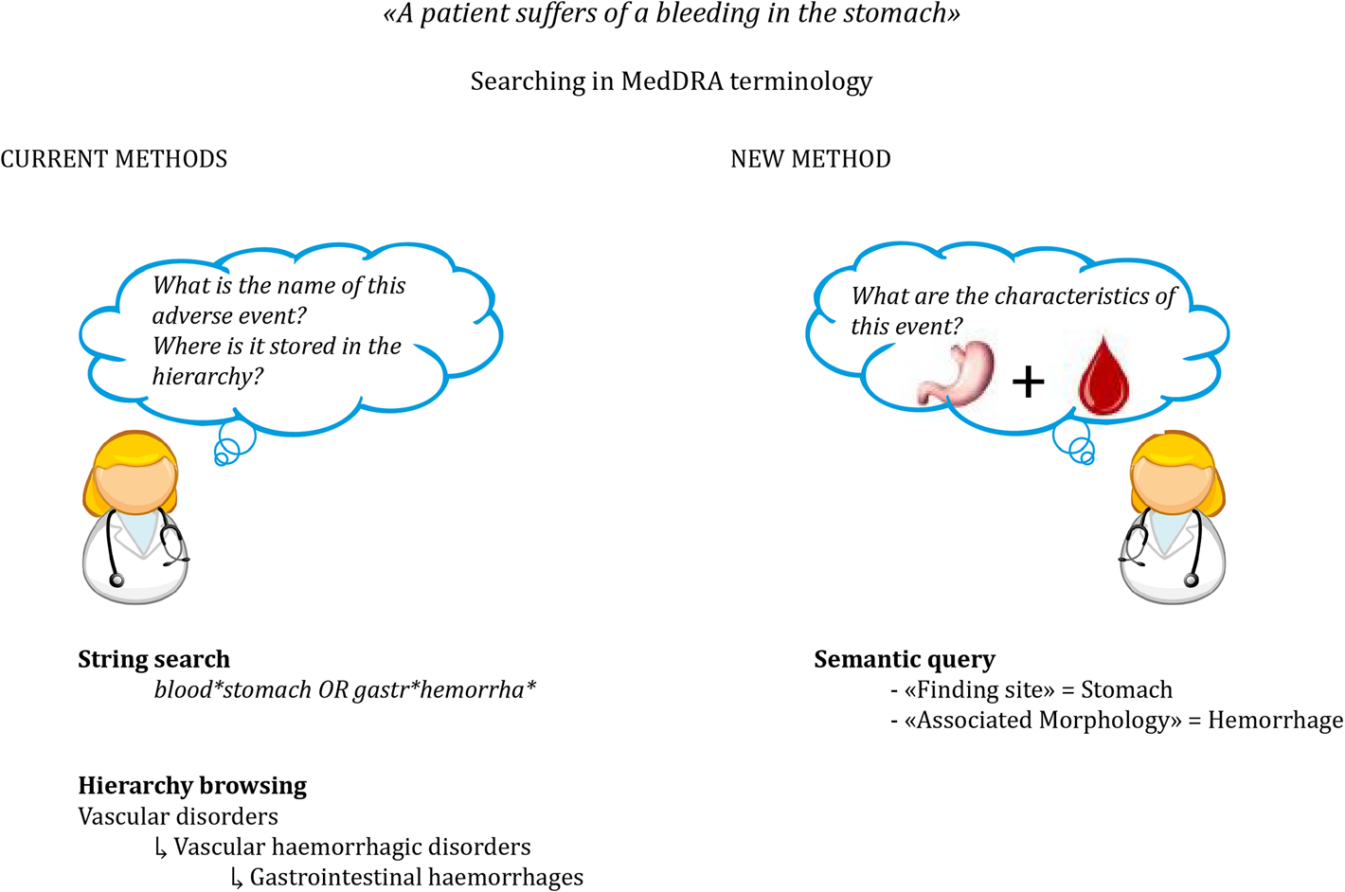
Comparison between methods to search in the MedDRA terminology (adapted from [11] with permission). Left, usual method: string search and hierarchy search. Right, search through semantic relations made possible by OntoADR (semantic query)

A semantic resource, OntoADR, that described MedDRA terms with semantic relations has been developed based on the SNOMED CT ontology during a former European project [12]. Instead of searching one by one all terms in the MedDRA terminology, PVS can search groupings of terms using semantic relations such as “hasFindingSite” to describe the anatomical site concerned by the ADR, or “hasAssociatedMorphology” to describe the abnormal morphologies related to the ADR (see Fig. 2). This way, PVS could identify and group all relevant MedDRA terms related to a given change on/in a site. Currently, 67% of MedDRA’s PTs are described with at least one defining relationship with SNOMED CT [12].

MedDRA and OntoADR have been made available to PVS through two tools based on different interaction modes: OntoADR query tool (OQT) whose interaction is based on forms, and Sparklis, based on guided query building in natural language. A third tool, Mister VCM (MVCM), based on an iconic language, links ADR directly to MedDRA terms without relying on OntoADR. The ultimate goal of the PEGASE project is to identify the tool that best reconciles usability (“the extent to which a product can be used by specified users to achieve specified goals with effectiveness, efficiency, and satisfaction in a specified context of use.” [13]) and expressiveness (the quantity and type of questions the system can answer [14]).

However, the literature on the usability of health technologies, whether information technologies or medical devices, shows that poor usability hinders their use, prevents users from taking full advantage of them and can bias the assessment of their impact. Indeed, the presence of usability problems (e.g. navigation problems, display fragmentation) increases users’ cognitive workload. Medical terminology-based interfaces are also affected by these usability problems [3]. As a result, users spend their cognitive and attentional capacities to navigate, find and retain information, leaving them with few cognitive and attentional resources to perform their main tasks [15]. In particular, expert activities such as pharmacovigilance case-finding require the cognitive and attentional abilities of PVS to be fully available, letting PVS concentrate and reason about solving the problems they face: if cognitive and attentional capacities are taken up by solving navigation or information gathering problems, they are less available for the actual pharmacovigilance task, and user performance is reduced. On the contrary, an improvement in the usability of a technology is associated with a lower cognitive load and an improved performance [16].

As the usability of the PEGASE tools is likely to affect their acceptability [17] and their performance, it is necessary to evaluate and improve their usability before releasing them and evaluating their impact. This is the purpose of formative usability evaluations reported in this paper.

## Objective

The purpose of this study is to evaluate, compare and improve, as much as possible, three interfaces using different approaches for facilitating the use of MedDRA by PVS: forms, structured query-builder and icons. The study relies on two rounds of formative usability evaluations for identifying the usability problems of each interface. This evaluation has no preliminary intention to make the three tools converge towards an identical, potentially ideal mode of interaction.

## Development and description of the tools

One of the main characteristics of the PEGASE project is that it strictly follows a user-centered design process, in which the needs and the characteristics of the end-users and the usability of the tools under development are the focus of the design activities at each step of the design process [13]. Following the recommendations of the user-centered design approach, the design process started with a review of scientific papers addressing the usability of keywords search technologies and with a cognitive work analysis of the PVS strategies to search for MedDRA terms [18]. The results of this first phase were used to adapt the three tools as far as possible to the context of the MedDRA terms search by PVS. The following subsections describe the three tools as they were before their formative usability evaluations.

### OntoADR query tool

OntoADR Query tool (OQT) is a web-based interface to query OntoADR semantic resource [11]. It uses forms to support the creation of queries based on properties extracted from SNOMED CT semantic relationships. Users can choose properties to be included in or excluded from their queries using a tree representing SNOMED hierarchy or using keyword-based search. The tool updates the query automatically for the addition or suppression of the selected terms. The MedDRA terms resulting from the constructed query are presented in a selectable list from which users can choose terms to export or to search case reports in a subset of FAERS (FDA Adverse Event Reporting System).

### Sparklis

Sparklis [19] is a query builder in natural language that allows people to explore and query semantic databases without any knowledge of the Semantic Web technologies (RDF, SPARQL) [20]. Sparklis is implemented as a Web client running entirely in the browser, which directly connects to a semantic database to retrieve query results and suggest query elements. It covers a large subset of the SPARQL 1.1 query language, including complex graph patterns and complex expressions. All query features can be combined in a flexible way. Results are presented as tables. A configuration panel offers a few configuration options to adapt to different databases.

Sparklis reconciles expressivity and usability in semantic search by tightly combining a Query Builder, a Natural Language Interface, and a Faceted Search system. As a Query Builder, it lets users build complex queries by composing elementary queries in an incremental fashion. An elementary query can be a class (e.g., “a film”), a property (e.g., “that has a director”), an entity (e.g., “Tim Burton”), a reference to another entity (e.g., “the film”), or an operator (e.g., “not”, “or”, “highest-to-lowest”, “+”, “average”).

As a Faceted Search system [21], at every step, the query under construction is well-formed, query results are computed and displayed, and the suggested query elements are derived from actual data - not only from schema - so as to prevent the construction of non-sensical queries or empty results. The display of results and data-relevant suggestions at every step provides constant and accurate feedback to users during the construction process. This supports exploratory search, serendipity, and confidence about final results [22].

As a Natural Language Interface, everything presented to users - queries, suggested query elements, and results - are verbalized in natural language, completely hiding SPARQL behind the user interface. Compared to Query Answering (QA) systems [23], the hard problem of spontaneous natural language understanding is avoided by controlling query formulation through guided query construction, and replaced by the simpler problem of natural language generation. The user interface lends itself to multilingualism, and is so far available in English, French, and Spanish.

As Sparklis is so generic, it required some data preparation and adaptations in order to be applied in the PEGASE project [24]. First, a semantic database was built by integrating the MedDRA terminology, the OntoADR and SNOMED ontologies, and patient data from FAERS. Second, Sparklis was extended to cope with the term hierarchies found in MedDRA and SNOMED. The latter introduces some complexity that may hinder the responsiveness of Sparklis, depending on the performance of the database server.

### MVCM

The MVCM tool [25] focuses on the use of the VCM iconic language for browsing MedDRA. VCM [26] is an iconic language for representing the patient main clinical conditions, including symptoms, diseases, physiological states (e.g. age class or pregnancy), risks and history of diseases, drug and non-drug treatments, lab tests and follow-up procedures. It includes a set of visual primitives (5 colors and 150 pictograms) and a simple grammar to combine these elements and create thousands of icons.

For representing clinical signs and disorders, a VCM icon is made of a color, a basic shape, a central pictogram and a set of modifier pictograms. The color indicates the temporal aspect of the icon: red for current states of the patient, orange for risk of future states, and brown for past states. The basic shape is a circle for physiological states or a square for pathological states (diseases and symptoms). The central pictogram indicates the anatomico-functional location (e.g. endocrine system) or the patient characteristic (e.g. pregnancy) involved; and special pictograms are available for the main specific disorders associated with a specific anatomico-functional location (e.g. diabetes for endocrine system). Modifier pictograms can be added to specify (a) a general pathological process (e.g. tumor, infection), and (b) a “transversal” anatomical structure that can be present in many anatomico-functional locations (e.g. blood vessels and nerves are present in most organs).

The mapping between MedDRA terms and VCM icons was achieved using the VCM ontology [27], which formalizes the semantics of VCM icons.

MVCM [26] is a schematic anatomical sketch that shows the 37 most generic VCM pictograms, grouped in 6 regions: the head, the hat (for social medicine), the thoughts (for psychology and addictions), the trunk, the arm (only one limb is detailed) and the etiologies (outside the body).

The MVCM interface for searching pharmacovigilance database is split in two parts (see an example in Fig. 6): the left part offers various methods for querying, and the right part displays the results. Three methods are proposed for searching: (a) full-text search by entering keywords, (b) iconic search by clicking a pictogram on MVCM, and (c) hierarchical selection by choosing the desired depth level in MedDRA using checkboxes. The three methods can be used independently or in combination, in any order. It also allows to select drugs and to start the search in the pharmacovigilance database.

The MedDRA terms found are grouped by VCM icons in small panels, each displaying at most five terms. The entire list of terms can be obtained by clicking on the panel. Moreover, the user can click on a term to display its parents and children, allowing a hierarchical navigation in MedDRA.

## Methods

Following the user-centered design process recommendations, the formative usability evaluations of the tools were performed in an iterative manner [13] with two rounds. First, a cognitive walkthrough (CW) was performed. Based on the design recommendations from this evaluation, the designers made modifications to their tools to improve usability and make them better fit to the needs of the PVS. Second, once this re-engineering phase was completed, usability testing was organized.

CW is a method developed to evaluate how easily a tool can be handled [28]. It focuses on the learnability of the tool for untrained or uncommon users. The CW proceeds through the analysis by experts in human factors of the mental processes required by users during the interaction with a tool [29]. It aims at identifying the design aspects of the system that may perturb the interaction of the user or even prevent her from using the tool.

Usability testing consists in observing representative end-users interacting with the technology under-evaluation to perform tasks [30, 31]. During the evaluation, users are commonly asked to “think-aloud” while interacting with the tool to get insights on their cognitive processes during the interaction. The analysis of behaviors and verbalizations of the participants enables to identify the problems they encountered with the technology and the usability defects that are behind [30].

Both methods have their own advantages and drawbacks. The literature recommends using these two methods together to improve the detection of usability problems [32]. Methods and results are presented as far as possible in accordance with reporting guidelines in the field [33].

### Formative cognitive walkthrough

Two researchers in human factors (LD and RM) conducted the CW. For each tool, the researchers first determined the sequence of tasks a user is intended to perform with the tool. Then, they examined the graphical user interface (GUI) state, before and after each action of the sequence of tasks, to assess whether PVS would succeed in performing the appropriate action at each point of the sequence. To do so, they asked systematically 4 questions at each step of the sequence [29]:

- Will the user be trying to achieve the right effect?
- Will the user notice that the correct action is available?
- Will the user associate the correct action with the desired effect?
- If the correct action is performed, will the user see that progress is being made?

The two researchers discussed each question until a consensus on the answer was reached. Each time a question was answered negatively at a point of the sequence of action, the reason was noted, the usability defect behind was searched and categorized according to usability heuristics [34], and a recommendation was proposed to fix this usability defect.

At the time of this evaluation, MVCM was not fully developed for pharmacovigilance. Thus, the CW was performed on screenshots of the different steps of the sequence of tasks. Descriptions given by the developer enabled to understand the GUI and the behavior of the tool at each point of the sequence.

The results were given to the designers of each tool along with videos of mock-ups illustrating recommendations for improvement. Then, designers analyzed those results to correct issues that may have affected the usability of the system and made the changes that were technically possible.

### Formative usability testing

**Fig. 3:**
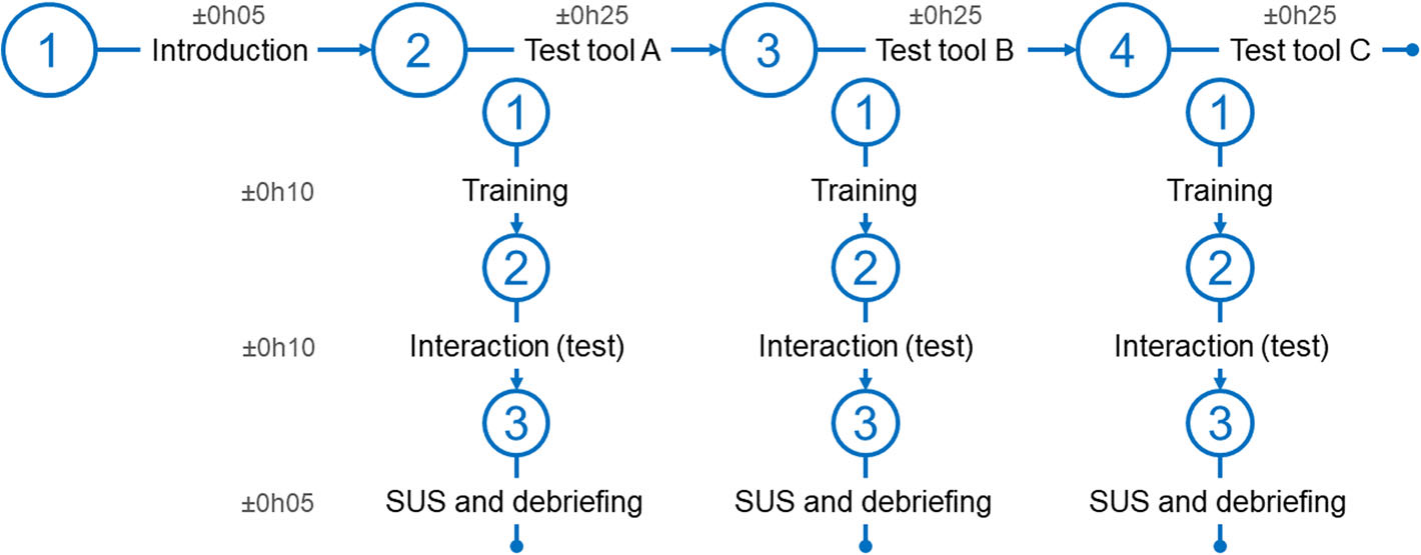
Overall organization of the usability tests. Time is expressed in hours and minutes

Once the three tools have been redesigned, a second round of evaluation was carried out with usability testing. Tests were performed individually on the French version of the tools (the GUI and the relations were in French; only the SNOMED CT terms were in English). Figure 3 represents the organization of a test session. After a short introduction to the PEGASE project and signature of consents, participants tested each tool successively. The running order of the tools’ tests was offset to prevent learning effects (Table 1).

**Table 1:** Order of the tools for each participant and question of pharmacovigilance answered at each test

	Sparklis	PV question	M. VCM	PV question	OQT	PV question
P1	A	3	B	6	C	1
P2	A	5	C	1	B	6
P3	B	2	A	3	C	5
P4	B	3	C	2	A	4
P5	C	2	A	5	B	3
P6	C	5	B	6	A	2

Letters (A, B, C) represent the order of the tools tested for each participant, the number (1 to 6), the question of pharmacovigilance answered at each test

For each tool, the participants were first introduced to the SNOMED CT terminology (its purpose, philosophy, and organization) with a focus on the semantic relations, and to the tool to evaluate (philosophy, use instructions, and descriptions of the main features). Then, a question to a PVS, randomly attributed, was given to each participant as both written text and verbal description. The questions were inspired from actual case reports in the scientific literature selected by a PVS to represent questions that pharmacovigilance centers may receive. These cases should not be too frequent or too obvious (i.e., not containing the corresponding MedDRA terms in their title or description) for the PVS to be unfamiliar with the MedDRA terms to be used and search for them using the tools. Moreover, since only 67% of the PTs are described with at least one defining relationship with SNOMED CT [12], it was important to check beforehand that the target terms for the cases were mapped to at least a minimal set of SNOMED CT concepts. The final 6 questions targeted were: (1) hearing disorder [35], (2) acute kidney injury [36], (3) progressive multifocal leukoencephalopathy [37], (4) glycemic dysregulation [38], (5) leukocytoclastic vasculitis [39], and (6) glaucoma [40]. In this way, these cases make it possible to test the main functions of the three tools.

Each participant was asked to use the tool to find case reports that may help answer the question and to think-aloud all along the interaction. In case of difficulty in finding a SNOMED term due to a lack of knowledge of English terms, participants were asked to express the searched term in French and the experimenter gave them the English version. Once the participants considered that they had found the case reports, they were asked to complete the System Usability Scale (SUS [41]). Each tool’s test ended with a debriefing on the interaction with the tool to deeply understand the participants’ behavior, and to get their opinion on the advantages, drawbacks and usefulness of the tool.

A total of 6 PVS were recruited. This sample size is in line with the recommendations from the literature and standards in the field (5 to 8 per user profile [42, 43]): 6 participants enable to detect more than 85% of the tools’ usability defects.

The tests were performed in the office of the PVS. During the tests, a human factors researcher sat further back the participants to observe their behavior. Data collected included the hesitations, misunderstandings, difficulties and usage errors along with the usability defects behind them, and participants’ verbalizations. Data were analyzed independently for each tool, to identify room for improving the usability. The tests were audio and video recorded.

## Results

The three tools do not have the same level of functionality and complexity. Therefore, they should not be compared based on the number of usability problems detected.

### OntoADR query tool

The results of the two rounds of usability evaluation are presented in Additional file 1: Appendix 1 along with the resulting suggestions for improvement. A total of 12 usability defects were uncovered with the CW. During the reengineering phase, either suggestions for fixing nine defects have been (partially) followed (nine cases), or the concerned feature has been removed. Usability tests uncovered nine problems: three ones were already detected by the CW, three ones which were not detected during the CW, and the three last ones concerning some new features.

The biggest issues concerned the use of SNOMED CT as PVS did not know this terminology. Indeed, SNOMED CT is very different from MedDRA. Even with training before the usability test, PVS did not understand its intrinsic logic and how to use it, thus using SNOMED CT was difficult for the participating PVS. Those issues with SNOMED CT were amplified by the fact that the SNOMED CT terminology was in English, by the long lists of (sometimes look-alike) items proposed in the editable drop-down menus and by the absence of organization of these items. During the usability test, human factors experts had to help PVS choose the right SNOMED CT relation or term to use.

Another important set of usability problems was related to the building of the query: in the first version of OQT, the visual organization of the page did not fit the cognitive processes underlying information search (steps were organized from right to left while information search proceeds from left to right), the use of the Boolean connectors was not explicit to the users, and several modes to build different types of queries co-existed on the GUI. Almost all the problems related to the query building disappeared after the reengineering.

Despite remaining usability problems, during the usability test, participants perceived the interaction with OQT as easy, as it was reflected by the 57 score on the SUS scale (“ok” tool according to [44]), and by positive comments on its ease of use to build a query. Due to the use of the SNOMED CT to build a query, participants found the query building process to search case reports too complex. This may be in part explained by the GUI and SNOMED CT being respectively in French and English, and SNOMED CT terms having a form that does not look like medical terms. Finally, participants regretted not being able to explore the MedDRA hierarchy into this tool to ensure that all relevant MedDRA terms were selected. Consequently, it was suggested to facilitate the use of the SNOMED CT to search and group MedDRA terms, especially by replacing those terms by actual medical terms and their synonyms in French. Without this evolution, it is unlikely the tool would prove to be used and useful.

**Fig. 4:**
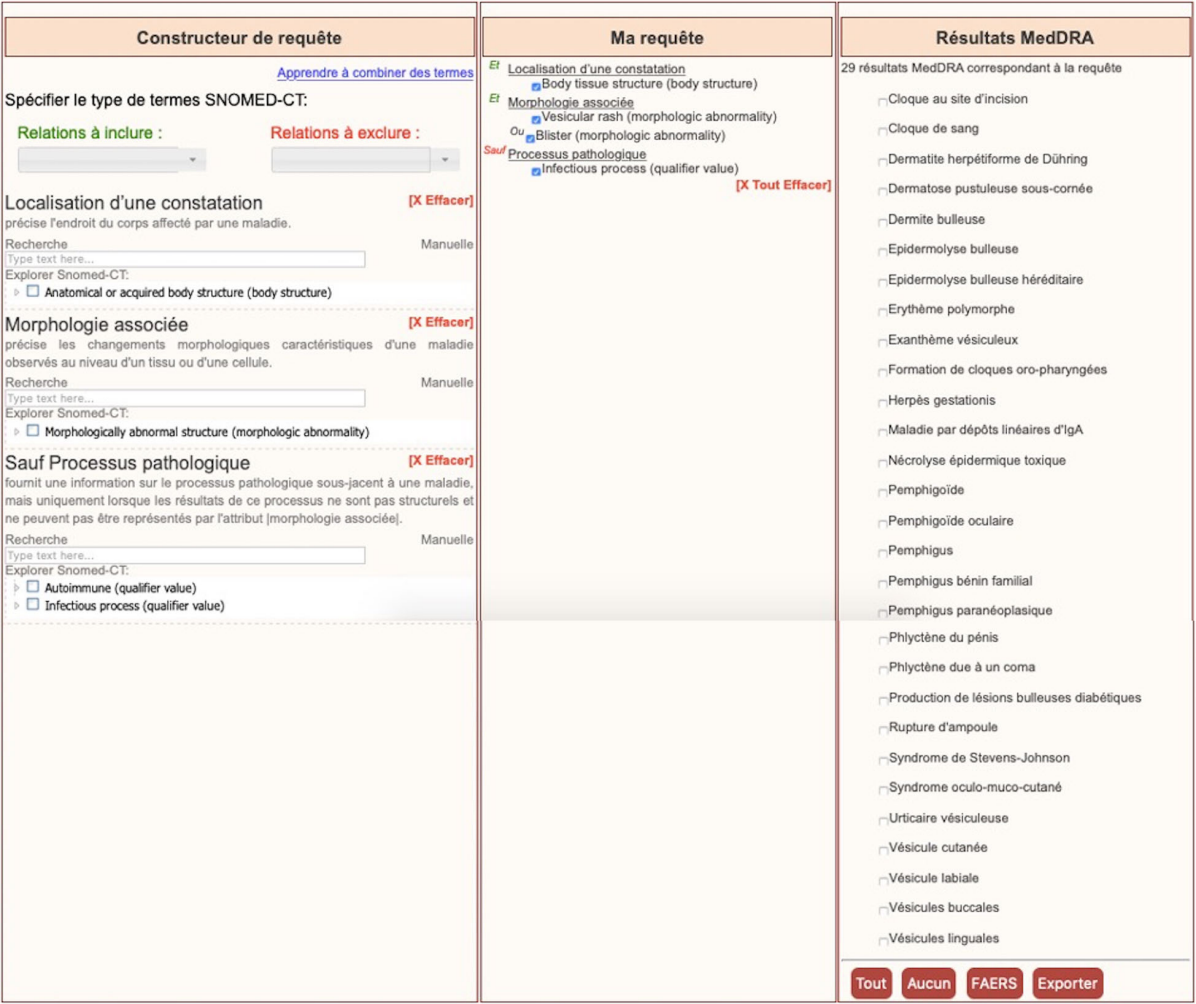
Screenshot of the OQT tool (version 2). The left frame contains the fields and trees needed to search properties for the construction of a query, the middle frame shows automatically the query being constructed and the right frame shows the MedDRA terms found for the current query. This last frame contains buttons allowing the user to select or deselect all terms, export selected terms or use them to search case reports in FAERS

The results of the usability testing were used by the developers to improve the OQT. The new version of the GUI is depicted in Fig. 4.

### Sparklis

The results of the two rounds of Sparklis evaluation are presented in Additional file 1: Appendix 2 with suggestions for improvement. A total of 14 usability problems were detected with the CW. Four problems identified through the CW could not be tested during the usability test session: due to test time limitations, very simple queries were used preventing from using some functions. The usability tests identified nine problems of which five had already been detected by the CW, and four new ones. It should be noted that technical problems (very long response time of Sparklis) strongly disturbed five of the six usability tests.

As for OQT, one of the main problems detected comes from the use of SNOMED CT semantic relations that PVS are not familiar with and from the use of the English SNOMED CT terminology which makes it more difficult to find the relevant terms. It was therefore recommended to make the use of SNOMED CT semantic relations invisible and to consider the use of a French version of the terminology.

Nevertheless, the main problem of Sparklis is related to the mode of interaction to build a query: it is very different from the mode the PVS are currently used to (e.g. in the BNPV). For example, the change of focus to build the query is not immediately understood by all users despite training. The same problem applies to the distribution of item categories and lists of terms in different menus. The lack of user guidance to know which menu to use hinders or even blocks users. Another problem is that Sparklis is a very general, powerful, tool that allows to formulate a very wide range of query structures: the task under evaluation is only one kind of the many possible searches. Yet, PVS usually perform a small number of query structures; therefore, they can get easily lost in the different possibilities. It is necessary that the version of Sparklis for PVS offers only the necessary and sufficient items to perform daily PV queries, with an expert mode that allows more possibilities for complex case reports. This option has already been partially integrated following the CW with the creation of different expert modes, but the novice mode needs to be further simplified to be used by beginners. More intensive training is needed to enable PVS to use Sparklis efficiently.

In terms of user experience, at first glance, participants were disturbed by the appearance of Sparklis, it seemed for them “difficult to use” and “complex”. However, after training, the participants said that they generally understood the principle of use, even though they believed they needed more explanation and training to use it correctly. This tool has been preferred by some participants in particular because its flexibility allows to make different types of requests, simple and complex, via MedDRA or SNOMED CT terms. Even if this flexibility makes it difficult for novice users to use the tool, it also increases its potential usefulness for current pharmacovigilance practice. The average SUS score of 43 (poor satisfaction [44]) at the end of usability tests seems to reflect mainly the annoyance of users due to Sparklis slowing down during the tests, and not an actual dissatisfaction regarding its functionality.

**Fig. 5:**
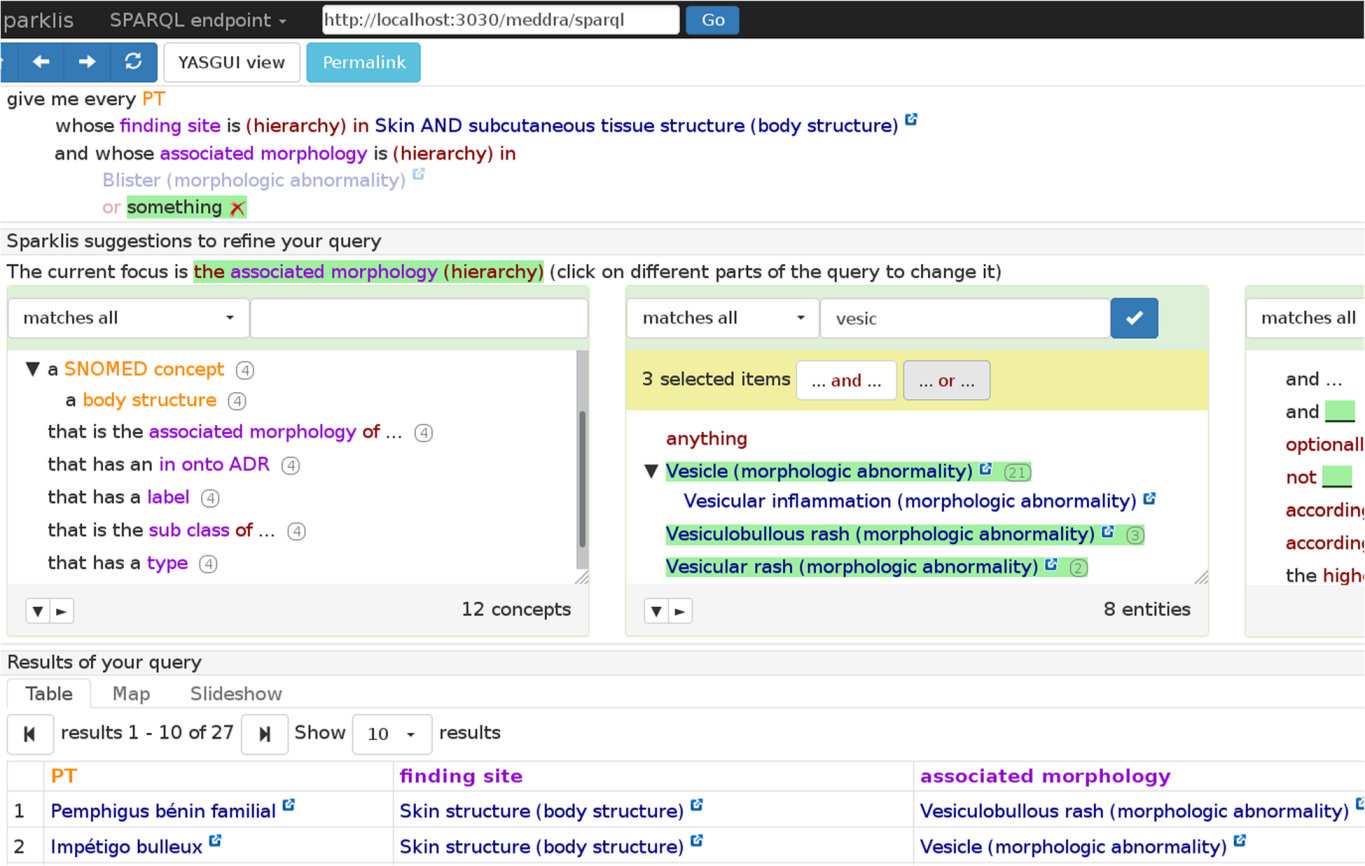
Screenshot of Sparklis on PEGASE data illustrating the process of building a query (version 2019–05). The current query (at the top) selects PT in MedDRA whose finding site is “Skin and subcutaneous tissue structure”, and whose associated morphology is various morphologic abnormalities. A first abnormality, “Blister”, has already been selected, and the user is in the process of selecting (at the center) a disjunction of three more abnormalities (“Vesicle”, “Vesiculobullous rash”, “Vesicular rash”). The keyword “vesic” was input at the top of the list of suggested terms in order to ease their retrieval among a long list of suggestions. Sparklis suggests changes to be applied to the current focus (here, the focus is on the associated morphology of the selected PT): at the middle left, Sparklis suggests types and relations to refine the query; at the middle center, terms denoting associated morphologies relevant to the query; and at the middle right, query modifiers and operators (e.g., “and”, “or”). The table of results of the current query is shown at each step. Here, it shows the selected PT along with their finding sites and associated morphologies

The results of the usability testing were used by the developers to improve the tool. The new version of the GUI is depicted in Fig. 5.^1^

### MVCM

The results of the two MVCM evaluation rounds are presented in Additional file 1: Appendix 3 along with suggestions for improvement. A total of five usability problems were detected with the CW; they were corrected during the re-engineering phase. The usability tests identified nine other problems.

MVCM generally follows the way PVS search for keywords. However, problems with the guidance and significance of the terms used can hamper users and impair search efficiency: some information and interface interaction elements are not sufficiently visible (e.g., “ back “, “ text search “, “ tooltips “) or intuitive (“ combination of several icons “, “ selection of level in the hierarchy “, “ adding a term to the query “).

In terms of their experience, participants found MCVM “fun” and “simple”. Participants who were resistant to the tool when MCVM was first presented, appreciated MCVM after use. According to the PVS, this tool would be most useful for people with little knowledge of pharmacovigilance to allow them to deal with simple case reports. Its usefulness would be more limited once the MedDRA terms are known or when ADR are not anatomically localized or are very specific (e.g., Progressive multifocal leukoencephalopathy). The SUS score of 72 (“good” [44]) shows a good level of satisfaction with this tool.

**Fig. 6:**
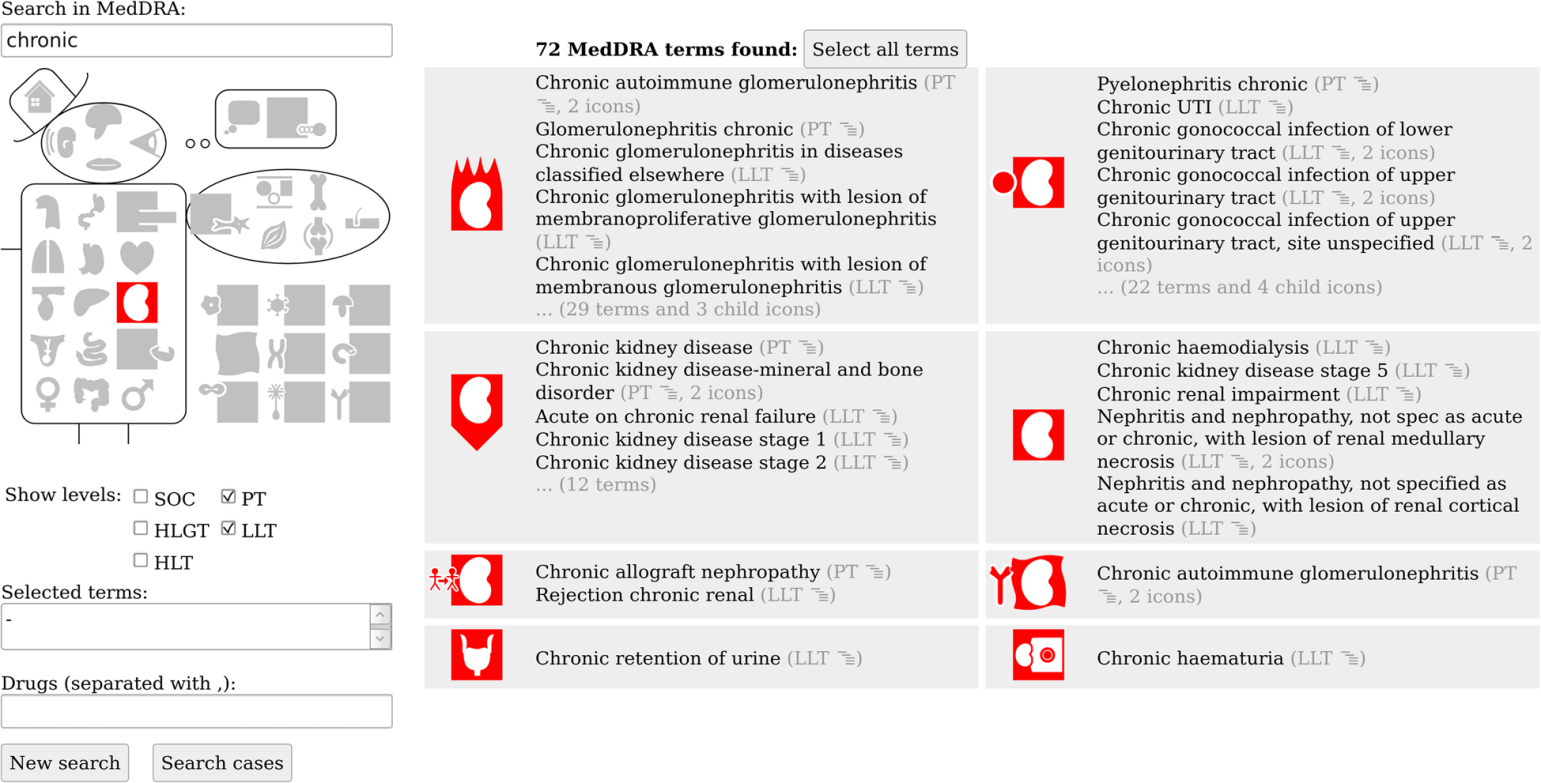
Screenshot of MVCM GUI (version 2.4). The screenshot presents the state of the GUI after searching for “urinary tract” (with VCM pictogram) and “chronic conditions” (with lexical keyword)

**Fig. 7:**
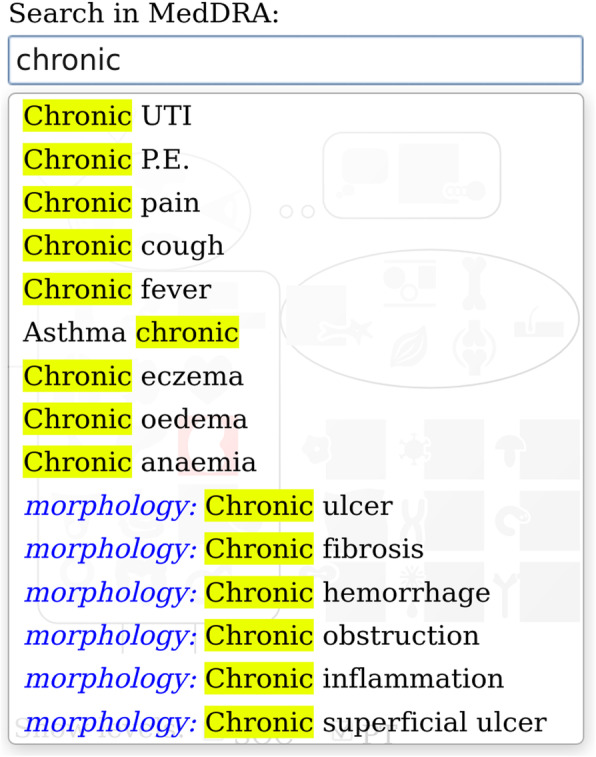
Screenshot of MCVM’s keyword search feature (version 2.4). The screenshot presents both textual keywords (in black) and semantic concepts from SNOMED CT (with the name of the relationship in blue)

The results of the usability testing were used by the developers to improve the tool. The new version of the GUI is depicted in Figs. 6 and 7.

## Discussion

### Principal results

The present study aimed to evaluate, compare and improve the usability of three tools developed to help PVS identify and group all relevant MedDRA terms using three different approaches: forms, structured query-builder, and icons. Two rounds of formative usability evaluation were performed: first with a CW, and then with a usability testing. A reengineering phase took place between both evaluation phases to improve the usability of the three tools.

All tools had usability problems during the first round of CW evaluations. Some problems were not resolved during the re-engineering phase either because usability testing was desirable to confirm them, or because these problems required too extensive modifications to the tools (by affecting the underlying philosophy of the tool), or because technical limitations did not allow the problem to be resolved. Other problems arose in the second round of evaluations because the interface had been modified (e.g., adding features) or because these problems were only detectable by observing real users interacting with the tools [32]. It should be noted that some problems detected with the CW method were not detected with the usability testing although they had not been corrected: these usability problems did not really bother users when interacting with the tools and can be considered as false positives which diminish the validity of the results (defined by Sears (1997) as a “focus on issues that are relevant” [45]) of the CW. Applying two complementary usability assessment methods allows for the weighting of the importance of the problems detected: those that really bother users should be addressed first.

The three tools cannot be compared in terms of the number of problems detected because they have different levels of complexity. Nevertheless, it is possible from a qualitative point of view to identify types of problems that were found in several tools.

First of all, although using SNOMED CT to find and group MedDRA terms seemed a promising idea when the PEGASE project started, the evaluations showed that the requirement that the user needs to understand this resource was an important limitation for Sparklis and OQT. Indeed, the French PVS are not familiar with SNOMED CT and its structure, and the short training did not allow them to understand and acquire the needed experience for the efficient use of semantic relations. On the contrary, MVCM does not rely on SNOMED CT for interactions with users and was well perceived by test participants. This suggests that interacting with SNOMED CT (e.g., using the appropriate semantic relation) might be the origin of the problem as it has been already pointed out [46]. Sparklis and OQT have to find a solution for improving interactions with SNOMED CT. One possible solution to overcome this problem would be to train and coach PVS more intensively to use SNOMED CT. However, even if training would allow PVS to master the main categories, it would not allow them to master the entire ontology (352,567 concepts [47]). As one PVS noted during a usability test, having to learn SNOMED CT, “just shifted the problem” from a lack of knowledge of MedDRA terminology as more training on MedDRA terminology would be more appreciated. The optimal solution would be to “hide” the use of semantic relations and SNOMED CT ontology from the PVS so that they benefit of their advantages without having to learn them. This solution requires developers to thoroughly rework how the tools work and can be a lengthy task. However, this “invisibility” of the interaction with SNOMED CT would certainly be appreciated by the PVS; indeed, the MVCM tool that does not show semantic relations of SNOMED CT has been appreciated for its simplicity.

The use of item lists to perform queries by OQT and SPARKLIS was also problematic because of the length of the lists and their non-intuitive organization for the PVS. This usability problem is frequently found in health information technologies [48] and also in terminology-based interfaces [4]. Even if this problem seems simple to solve, it nevertheless requires a reflection on the items to be kept in the lists, which may also question the way to carry out the queries. Indeed, the calibration of the lists is necessarily done according to the profile of the users, but novices and experts have different needs to perform a query; lists calibrated for a single profile would make the tools useless for the other profiles.

The other noteworthy usability problems were related to the GUI that were not guiding enough, i.e., their organization and the icons and labels used did not always sufficiently inform participants about what they could do and how. These usability problems are also commonly found in health technologies [48, 49] and in terminology-based interfaces [3, 4, 50–53]. To be corrected, these problems require work to reformulate information on labels and buttons, which only requires modifying the interface, not the tool in-depth, and can be done quickly.

Even if the three tools cannot be quantitatively compared in terms of usability, feedback from participants during the tests made it possible to identify the perceived advantages and disadvantages of each tool (see Table 2). In fact, the perceived usefulness of the tools depends on the complexity of the MedDRA term search. For the most obvious and common terms to be found, the MVCM tool, the icon-based interface, would appear to be more useful due to its ease of use. On the contrary, for the less frequently used MedDRA terms or those distributed in several SOC, Sparklis, the structured query-builder based on natural language, would seem to be preferable thanks to its great power and flexibility, the latter having proven their usefulness in other domains [19]. OQT, the form-based interface, would seem to be a good compromise between both tools. However, the necessary knowledge of the SNOMED CT logic could prevent the routine use of Sparklis and OQT by PVS who are not experts in this terminology.

**Table 2:** Synthetic list of advantages and drawbacks of the three tools under evaluation

	OQT (forms)	Sparklis (natural language)	MVCM (iconic language)
Advantages	• Quickly shows the results of term searches • Easy to use	• Powerful tool adapted to different kind of practices • Single page for the query and case analysis which facilitates the refinement of the query	• Intuitive and “playful”, visual iconic language • Sense of familiarity: no need to go through SNOMED CT • Possibility to navigate in the MedDRA hierarchy
Drawbacks	• Requires knowledge of SNOMED CT logic • Requires knowledge of the right SNOMED terms and their hierarchy • Not possible to search/navigate in the MedDRA hierarchy	• Requires knowledge of SNOMED CT logic • Requires knowledge of the right SNOMED terms and their hierarchy • Requires practice to fully understand how to use the tool	• Limited in possible searches • Not suitable for expert PVS

### Strengths and limitations of the formative evaluations performed

Although many studies have focused on software based on standardized terminologies, few have actually evaluated the usability of these technologies to improve it [3, 4, 50–53]. In the context of the use of MedDRA terminology, to our knowledge only one study has evaluated its usability ([11] on OQT). However, in this study the usability evaluation was only carried out subjectively using the SUS questionnaire; even if the latter enables to assess the level of satisfaction of the participants, it does not help in understanding what the users liked or disliked, and what features of the tools may have disturbed the performance of their tasks. The present study provides data to improve user interaction by concretely evaluating and comparing the usability of three different types of tools allowing the exploration of a medical terminology.

The main strength of the study is that it has combined two different methods in order to overcome the shortcomings of each of them. The CW on the first versions of the tools allowed to detect so-called common problems in usability, guidance and intuitiveness. Then, the usability testing allowed to detect usability problems linked to the mismatch between the work models implemented in the tools, and the tasks actually performed by the PVS [32]. In addition, iteratively performing two evaluation rounds also made it possible to detect new usability problems caused by interface modifications.

Furthermore, for the evaluation of MVCM, usability testing compensated for the potential weakness of the CW performed on screenshots by detecting problems that the CW may not have allowed to be identified.

Several points may have affected the completeness of the problems detected. Due to the slowdown of Sparklis, the behavior of the PVS may not have been natural and some problems that would have emerged in real life may not have been detected. During the tests, participants interacted with an interface and relationships in French while the terms to be used were in English. Constantly switching from French to English may have hindered their interactions with the tools, also hindering the detection of potential problems. Furthermore, in order not to extend the duration of the tests (that were approximately 1 h30 long), it was chosen not to test some secondary functions but to concentrate the evaluation on the main functions for which usability problems could hinder or block users. It is more important to detect problems that limit optimal use and that will deteriorate user performance rather than to draw up an exhaustive list of all problems, even those very rarely encountered and with no impact. Users were only able to make two or three queries per tool, which limited appropriation.

The target terms of the cases used during the tests should be mapped to at least a minimal set of SNOMED CT concepts. Yet, there were still deficiencies in some definitions of MedDRA terms in OntoADR. This kind of deficiency would have impacted studies on the completeness of the underlying knowledge representation of MedDRA terms. The present study focused on the usability of the GUI of the tools. It is unlikely that those deficiencies in the mapping had consequences for users and influenced the results of the evaluation. Nonetheless it would make it worse if new examples with no available SNOMED CT definition were selected in future work. This is the reason why improving completeness of definitions in OntoADR is important to guarantee performances in real life that are close to performances we observed in constrained laboratory environment.

### Future work

The corrections related to usability problems that were feasible during the period of the project while respecting the mode of interaction of the tools were performed. The next step consists in comparing the expressiveness of the output of the three tools as well as their acceptability during an in-situ evaluation. This way, the tools can be really compared on their mode of interaction, their expressiveness and their perceived usefulness. The perspectives of this work also include the adaptation of the three tools to other medical terminologies, e.g. for supporting the coding of electronic health records.

In addition to the context of answering questions from healthcare professionals, OntoADR could be useful in pharmacovigilance to analyze with natural language processing textual resources such as comments associated with some case reports, or to perform automated signal detection. In a separate study, we performed some tests on signal detection. Although it is possible to increase the number of detected signals by grouping terms with [54] or without [55] an ontology compared to performing signal detection with single PTs, we observed that grouping does not necessarily allow to detect these signals earlier and had to figure in which situations grouping may expedite signal detection. We assume that groupings in our experiments consisted of too large number of terms which was not appropriate: focusing on smaller sets of MedDRA terms could lead to better results. Further studies need to be conducted to test this hypothesis.

## Conclusion

Three tools with different kinds of interface have been developed or adapted to help PVS find MedDRA terms and group them together to perform pharmacovigilance queries. In order to improve the usability of the tools as much as possible, two iterations of formative evaluations have been carried out through CW and usability testing. These two methods proved to be complementary by detecting different problems. The results of these two iterations made it possible to identify the strengths of each of the tools but also their weaknesses in order to address them before evaluating their acceptability by the PVS along with the expressiveness of their results. The perceived usefulness of the three kinds of tools depends on the complexity of the MedDRA term search to perform.

## Supplementary information

**Additional file 1: Appendix 1.** Usability defects detected in OQT through the CW and the usability testing sorted according to Scapin and Bastien usability category [34]. Slashes represent no change for a problem. Hyphens represent no detection of a problem. **Appendix 2.** Usability defects detected in Sparklis through the CW and the usability testing sorted according to Scapin and Bastien usability category [34]. Slashes represent no change for a problem. Hyphens represent no detection of a problem. **Appendix 3.** Usability defects detected by the CW and the usability testing for MCVM sorted according to Scapin and Bastien usability category [34]. Slashes represent no change for a problem. Hyphens represent no detection of a problem.

## Data Availability

All data generated or analyzed during this study ((list of usability problems per tool and per evaluation rounds are available in appendices) are included in this article’s supplementary information files (appendices).

## Abbreviations

ADR: Adverse drug reaction
CW: Cognitive walkthrough
FAERS: Food and drug administration adverse event reporting system
GUI: Graphical user interface
MedDRA: Medical dictionary for drug regulatory activities
MVCM: Mister VCM
OQT: OntoADR query tool
PEGASE: *Pharmacovigilance Enrichie par des Groupements Améliorant la détection des Signaux Émergents*, in English improved pharmacovigilance and signal detection with groupings
PVS: Pharmacovigilance specialists
SNOMED CT: Systematized nomenclature of medicine - clinical terms
SOC: System organ class
HLGT: High-level group terms
PT: Preferred term
LLT: Low-level term
HLT: High-level term

## Footnotes

1 A screencast of the whole query building is available at http://www.irisa.fr/LIS/common/documents/pegase2018/#ExtraCase
