# Supplementary material for: How to interact with medical terminologies? Formative usability evaluations comparing three approaches for supporting the use of MedDRA by pharmacovigilance specialists
Source: BMC Med Inform Decis Mak. 2020 Oct 9;20:261. doi: 10.1186/s12911-020-01280-1 (PMC7547416; doi:10.1186/s12911-020-01280-1)
Supplement: Supplementary file 1 — Additional file 1: Appendix 1. Usability defects detected in OQT through the CW and the usability testing sorted according to Scapin and Bastien usability category [34]. Slashes represent no change for a problem. Hyphens represent no detection of a problem. Appendix 2. Usability defects detected in Sparklis through the CW and the usability testing sorted according to Scapin and Bastien usability category [34]. Slashes represent no change for a problem. Hyphens represent no detection of a problem. Appendix 3. Usability defects detected by the CW and the usability testing for MCVM sorted according to Scapin and Bastien usability category [34]. Slashes represent no change for a problem. Hyphens represent no detection of a problem. [file 12911_2020_1280_MOESM1_ESM.docx]

Appendix1. Usability defects detected in OQT through the CW and the usability testing sorted according to Scapin and Bastien usability category [34]. Slashes represent no change for a problem. Hyphens represent no detection of a problem.

|  |  | Round 1 - Cognitive walkthrough | | Reengineering phase 1 | Round 2 - Usability testing | | Reengineering phase 2 |
| --- | --- | --- | --- | --- | --- | --- | --- |
|  | Usability category | Usability problem | Suggestion for improvement | Actions made | Usability problem | Suggestion for improvement | Actions made |
| #1 | Guidance | Terms in the drop-down menus and lists of results are numerous and not sorted. | Keep in the list only the items that might be used by the PVS. Sort the items according to their use frequency. | none | The large number of items in the drop-down menus bother the users; they are confused by terms looking the same. | Keep only useful items for the users. For the SNOMED-CT terms, add their hierarchical level so that PVS could distinguish them from each other. | SNOMED-CT terms are restricted to those used in OntoADR.  Users will be trained. |
| #2 |  | - | - | - | The drop-down menus to select the “SNOMED-CT relation” to include or exclude are editable but this feature is not visible enough. Most of the participants do not use it to limit the number of items in the menu. | The editable part of the drop-down menu should look like an actual entry field. | None |
| #3 |  | The GUI does not inform about the use of Boolean operators to build the query. | Add a short tutorial explaining the use of the Boolean operators. | Implemented | - | - |  |
| #4 |  | The built query does not inform about the Boolean operators that are used. | Display the Boolean operators used in the query. | Implemented |  |  |  |
| #5 |  | The button to save a query is under the list of terms. When numerous terms are listed, the button is not visible enough. | Change the position of the button and highlight it to be more visible and always at the same place. | Feature removed | - | - |  |
| #6 |  | There is no feedback informing that a query is saved. | A pop-up message should appear once the query is saved. | Feature removed | - | - |  |
| #7 |  | Deleting a running query is only possible by clicking on the tab of the current query. | Add “close” icons next to the query, enabling deleting it easily. | Implemented | - | - |  |
| #8 |  | To combine several saved queries (to build complex queries), the user must click on the tab “Boolean query” which is not intuitive | Enable the user to build “complex queries” from the same tab as for short queries. | Feature removed | - | - |  |
| #9 |  | - | - | - | The button to delete a relation from the query has not been found by all participants. | Highlight this button. | The link is highlighted in yellow |
| #10 |  | - | - | - | The button to access to the page where the case reports can be found is not understood (“FAERS”). | Change its name into “see the cases”. | Label changed |
| #11 | Workload | Even if the tool is dedicated to building queries, the first page the user sees is not to build queries; the user has to explore a first page and click on the “query” tab. | At the tools’ opening, directly display the “building query” page. | Implemented | - | - |  |
| #12 | Compatibility | PVS are used to use MedDRA terms, not SNOMED-CT terms. They do not even know about SNOMED-CT. However, the whole tool, per see, works with SNOMED-CT terms. | The occurrence of this problem must be tested with actual users after a training to SNOMED-CT.  Nonetheless, end-users should be trained to SNOMED-CT. | Training implemented | The users do not understand the concept of the “SNOMED-CT relations”. They found it more complex than the MedDRA hierarchy concepts. | Do not explicitly use SNOMED-CT (hide it from the user’s view). | SNOMED-CT terms are restricted to those used in OntoADR.  Users will be trained. |
| #13 |  | The tool is in English while PVS are used to work in French. | Translate the whole interface in French. | Partially implemented | Using SNOMED-CT in English makes it more difficult to find the relevant terms. | Do not explicitly use SNOMED-CT (hide it from the user’s view). | The French SNOMED-CT is used. |
| #14 |  | - | - | - | PVS have no other choices than using the SNOMED-CT terminology to find MedDRA terms. Yet, for common queries, they already know the MedDRA term to use. | Add a “search by MedDRA term” feature. | This functionality is already available in the first version of OQT before the CW, and may be implemented in the new version. |
| #15 | Protection against errors | - | - |  | Clicking on a term in the SNOMED-CT explorer section added it to the query. | Display hierarchy explorer on demand to avoid accidental explorations. | By default, the hierarchy explorer is hidden.  Adding a term requires now to tick a box, and not on the term anymore |
| #16 | Significance of codes | Some labels are unusual to PVS (ex. “criterion” of the SNOMED-CT). | Revise the labels based on the habits and knowledge of the PVS. | Implemented | - | - |  |
| #17 |  | - | - | - | The message saying there is no case reported for a couple drug – MedDRA term is ambiguous. | Make it simpler. Write “0 cases with the term and this drug”. | A message has been added informing that the query with the selected terms has no results. |
| #18 | Consistency | On the “query” tab the list of resulting MedDRA terms is automatically displayed and updated while in the “Boolean query” tab, the user must click on the “calculate” button to get the list of MedDRA terms. | The “Boolean query” feature should work as the simple “query” feature. | Feature removed | - | - |  |

Appendix 2. Usability defects detected in Sparklis through the CW and the usability testing sorted according to Scapin and Bastien usability category [34]. Slashes represent no change for a problem. Hyphens represent no detection of a problem.

|  |  | Round 1 - Cognitive walkthrough | | Reengineering phase 1 | Round 2 - Usability testing | | Reengineering phase 2 |
| --- | --- | --- | --- | --- | --- | --- | --- |
|  | Usability category | Usability problem | Suggestion for improvement | Actions made | Usability problem | Suggestion for improvement | Actions made |
| #1 | Compatibility | The task model of the "query construction" (use of focus to add or remove an element, choice of elements searched for) does not correspond to the PVS logic. | Make the handling of the query more understandable by using a vocabulary and a structure that will be recognized by the PVS. By default, propose a query to be completed to search for case reports in the database. | Partially implemented and training implemented | Participants do not understand the phrasing of the query ("too much literature") and do not detect the errors in it. Once understood, the phrasing of the query is appreciated.  Despite the training, some participants do not notice that the focus can be changed. Once explained, they can do it again. | More in-depth training on the different stages of query handling. | / |
| #2 |  | The use of SNOMED-CT semantic relations is not usual for PVS. | The occurrence of this problem must be tested with actual users after a training to SNOMED-CT. Nonetheless, end-users should be trained in SNOMED-CT. | Training implemented | Participants found it more difficult to break a term down into sub-concepts to find the semantic relationships than to search directly for the MedDRA term.  Action was required to help PVS find the relationships to use for each query.  The level of the SNOMED-CT terms in their hierarchy is not known which led to errors. | Do not explicitly use SNOMED-CT (hide it from the user’s view). | Not possible to hide SNOMED-CT. Sparklis allows to use MedDRA only.  The set of SNOMED terms is now restricted to those related to MedDRA, and French labels have been added. |
| #3 |  | To judge the relevance of a MedDRA term for a query, in their daily work, the PVS examine the number of case reports coming out with this MedDRA term and compare it with their expectations. In the interface, the number is only shown on a sample of the database, which makes it impossible to judge correctly the relevance of a MedDRA term. | Automatically display the full number of case reports related to a MedDRA term next to that term in the lists of terms provided.  Indicate the total number of case reports when a term is added to the query. | Partially implemented | - | - | Display of results as nested tables, with a single row per case report. |
| #4 |  | The user cannot navigate through the MedDRA term hierarchy from a selected term. | Add the ability to navigate through the hierarchy via a context menu which appears when the user clicks on a MedDRA term. | When the user puts focus on a selected term in the query, the center box shows the hierarchy above and under the term | *Not tested* |  |  |
| #5 | Guidance | The users have 3 menus at their disposal to feed the query. Even if their functions are different, these menus are not distinguished by their appearance or by their titles. | Visually differentiate the 3 menus and give them headings that correspond to their content (e.g., properties and suggestions of...). | Partially implemented | The left and center menus are often confused. After further explanations all participants remember the functions of both menus. | A better visual differentiation between the columns is desirable (e.g. use a color code to distinguish the two boxes, these colors should correspond to the query elements that these boxes allow you to select). | This color coding already appears through the contents of each box.  Orange, purple and cyan in the left box; blue and green in the center box. |
| #6 |  | - | - | - | On the contrary to MedDRA terminology, SNOMED-CT is not hierarchical which disturbed participants who did not know “which level” they were at. | Do not explicitly use the SNOMED-CT (hide it from the user’s view). | Not possible to hide SNOMED-CT. Sparklis allows to use MedDRA only.  The set of SNOMED terms is now restricted to those related to MedDRA, and French labels have been added. |
| #7 |  | The third menu offers connectors (e.g. AND, OR, NOT). This menu is indistinguishable from the other menus, whereas the connectors perform very different functions from the contents of the other menus. | Delete the third menu and use instead a drop-down menu with the commonly used connectors (AND OR NOT) directly in the query. | Partially implemented | *Not tested* |  |  |
| #8 |  | The request is not highlighted which makes it difficult to be identified. | Highlight the query on the screen (e.g. font size, frame). | Partially implemented (put in bold) | - | - |  |
| #9 |  | The semantic relationships and terms displayed in the menus are very numerous. | Reduce the number of items by displaying only those that are relevant to the current practice of PVS.  Sort items by frequency of use.  Give the possibility to display more properties for the building of a less common query. | Implemented | - | - |  |
| #10 |  | The user can filter the proposals via a text field. The latter is not incentive, it looks like a button and not a search bar. | Make text fields look like text fields. | / | - | - |  |
| #11 |  | The search for SNOMED-CT terms must be carried out in English unlike the rest of the interface but this is not indicated to the user. | Make it appear in the text field that English must be used. | / | Several participants had to be told to write the terms in English and translate them to them. | Make it appear in the text field that English must be used. | French labels have been added for SNOMED-CT. |
| #12 |  | To add several terms to the query the user must press the "control" key on the keyboard and then click on the desired terms on the screen. This is not intuitive. | Allow to select one or more terms on the screen, then allow to add them to the query via an "add to query" button. | / | *Not tested* |  | Multi-select button are added. |
| #13 |  | - | - | - | A participant is unable to delete a term added to the query. | Provide a help function and more in-depth training on the different stages of query handling. |  |
| #14 | Significance of codes | Some icons and signs used to represent functions are not understandable (+) or too small to be seen and used (unfold). | Use common signs and icons to represent interaction possibilities. Give them a reasonable size to be visible and usable. | / | *Not fully tested* |  |  |
| #15 |  | Some of the terms used are not intuitive (e.g., "anything" to say that there is no case). | Clearly explain to the user why there are no cases (e.g. "There are no case reports in the database with a drug starting with "para" associated with the other elements of your query". Otherwise display the results for “para” with 0 indicated in the facets: "Paracetamol (0)". | / | Participants did not understand "anything" and either thought that they had entered the wrong term or that the database was incomplete. | Instead of not displaying terms because there are no case reports associated with the query already formulated, it would be better to display them in gray (at the end of the list of suggested terms) to indicate that they exist but are not selectable. When there are no terms to suggest, explicitly display that there are "0 results" rather than "anything". | « no element » is displayed in case of no match with case reports along with a few terms that match but for which there are no query answer. |
| #16 |  | The naming of semantic relations in the lists coming from SNOMED-CT (e.g. observation site) is not usual for PVS. | Adapt the names to the concepts used by the PVS | Implemented | - | - |  |
| #17 | Protection against errors | - | - | - | After entering a term in a text field, participants pressed "enter" to see suggested terms. This added directly the term to the query without them noticing it. | Do not allow a term to be added to the query if it has not been selected in the suggested terms. | Keystroke enter are used to trigger keyword search, rather than insertion in query. |
| #18 | Feedback | - | - | - | After clicking on a term in a list, some participants did not see that the term had been added to the query. | A visual feedback as an animation would help user see the term has been added. | Animations are added to attract user attention |

Appendix 3. Usability defects detected by the CW and the usability testing for MCVM sorted according to Scapin and Bastien usability category [34]. Slashes represent no change for a problem. Hyphens represent no detection of a problem.

|  |  | Round 1 - Cognitive walkthrough | | Reengineering phase 1 | Round 2 - Usability testing | | Reengineering phase 2 |
| --- | --- | --- | --- | --- | --- | --- | --- |
|  | Usability category | Usability problem | Suggestion for improvement | Actions made | Usability problem | Suggestion for improvement | Actions made |
| #1 | Consistency | The "search FAERS" and "new search" buttons are respectively to the left and right of each other. However, the search buttons are generally on the right. | The "search FAERS" button should be to the right of the "new search" button | Implemented |  |  |  |
| #2 | Significance of codes | The title of the "FAERS search" button is not understandable for French PVS who are not familiar with the FAERS database. | Change the title to "search for cases" | Implemented |  |  |  |
| #3 |  | Some of the icons used are arbitrary. Nothing in the interface allows PVS to know what these icons represent. | Describe the icons in a tooltip when the mouse passes over them. | Implemented |  |  |  |
| #4 |  |  |  |  | Adding a term to the query is not intuitive for all participants: some click on the name of the term rather than clicking on "+" because "+" is used in the usual PV software to unfold the hierarchy. Others do not see how a term can be added. | Add a term to the query by clicking on the term and access the hierarchy by pressing the "+". | Implemented; the “+” button is now replaced by another button that displays the hierarchy. |
| #5 | Compatibility | To judge the relevance of a MedDRA term to a query, PVS look at the number of case reports associated with that MedDRA term and compare it to their expectations. In the interface, the number of case reports associated with an icon representing a MedDRA term is not indicated. | Indicate the number of case reports associated with the icon representing the term MedDRA. | Implemented (in popup bubble) |  |  |  |
| #7 | Guidance | The text fields for drug names and keywords do not provide suggestions. | Add suggestions when entering keywords and drugs. | Implemented | - | - |  |
| #8 |  |  |  |  | Participants did not understand why some drugs were duplicated in the list of suggestions and what the difference between these duplicates was. | Check the base of the drugs to avoid duplicates. | Implemented |
| #9 |  | - | - | - | The fact that several icons can be combined (e.g., an organ and a type of infection) was not understood by some participants despite the presentation of the tool. | A more complete training will allow participants to become familiar with the icons and their meaning, the combination of icon/icon and text/icon. | A training software is being developed for VCM and the MVCM tool. |
| #10 |  | - | - | - | The text search is not identified by all participants. | A more complete training will allow participants to become familiar with the icons and their meaning, the combination of icon/icon and text/icon. | In addition, help labels are shown at the initial display of the interface. They occupy the space devoted to the search results and disappear after the first search is performed. |
| #11 |  | - | - | - | When changing levels in the hierarchy, the initial term is lost; participants get lost in the levels. | Display the hierarchy levels of the terms (LLT, PT...)  Keep displaying the sub-levels of the hierarchy (LLT, PT...) when selecting a higher-level term. | Implemented |
| #12 |  | - | - | - | The "back" function is not sufficiently visible: some participants use the browser function or cancel a search in progress by deselecting an icon they had clicked on. | Position the "back" button in a space at the top of the screen (above "type keyword(s) and enter"). If necessary, propose a "redo" button next to it that would allow to cancel this "back". | Implemented; the “redo” button is called “forward”. |
| #13 |  | - | - | - | The selection function for the level of the MedDRA hierarchy is not seen by all participants. | By default, preselect only PT. | Implemented |
| #14 |  | - | - | - | The tooltips were not all seen by the participants which led to misunderstanding of the icons. | Make the tooltips larger with more contrast. | Implemented |
| #15 |  | - | - | - | The way to display the hierarchy for a term is not found by a participant. | Display the hierarchy of a term by clicking on "+". | Implemented |
